# Long-Term Monitoring of Fecal Steroid Hormones in Female Snow Leopards (*Panthera uncia*) during Pregnancy or Pseudopregnancy

**DOI:** 10.1371/journal.pone.0019314

**Published:** 2011-05-02

**Authors:** Kodzue Kinoshita, Sayaka Inada, Kazuya Seki, Aiko Sasaki, Natsuki Hama, Hiroshi Kusunoki

**Affiliations:** 1 Faunal Diversity Sciences, Graduate School of Agriculture, Kobe University, Nada-ku, Kobe, Japan; 2 Kobe Municipal Oji Zoo, Nada-ku, Kobe, Japan; 3 Tama Zoological Park, Hino-shi, Tokyo, Japan; Texas A&M University, United States of America

## Abstract

Knowledge of the basic reproductive physiology of snow leopards is required urgently in order to develop a suitable management conditions under captivity. In this study, the long-term monitoring of concentrations of three steroid hormones in fecal matter of three female snow leopards was performed using enzyme immunoassays: (1) estradiol-17β, (2) progesterone and (3) cortisol metabolite. Two of the female animals were housed with a male during the winter breeding season, and copulated around the day the estradiol-17β metabolite peaked subsequently becoming pregnant. The other female was treated in two different ways: (1) first housed with a male in all year round and then (2) in the winter season only. She did not mate with him on the first occasion, but did so latter around when estradiol-17β metabolite peaked, and became pseudopregnant. During pregnancy, progesterone metabolite concentrations increased for 92 or 94 days, with this period being approximately twice as long as in the pseudopregnant case (31, 42, 49 and 53 days). The levels of cortisol metabolite in the pseudopregnant female (1.35 µg/g) were significantly higher than in the pregnant females (0.33 and 0.24 µg/g) (*P*<0.05). Similarly, during the breeding season, the levels of estradiol-17β metabolite in the pseudopregnant female (2.18 µg/g) were significantly higher than those in the pregnant females (0.81 and 0.85 µg/g) (*P*<0.05). Unlike cortisol the average levels of estradiol-17β during the breeding season were independent of reproductive success.

The hormone levels may also be related to housing conditions and the resulting reproductive success in female leopards. The female housed with a male during the non-breeding season had high levels of cortisol metabolites and low levels of estradiol-17β in the breeding season, and failed to become pregnant. This indicates that housing conditions in snow leopards may be an important factor for normal endocrine secretion and resulting breeding success.

## Introduction

Wild snow leopards (*Panthera uncia*) inhabit in highlands with rugged terrains. Their natural habitat makes their field observations extremely difficult, especially reproductive biology [1], [2], and hence, study of snow leopards in the wild is markedly less than in other felid species [3]. In addition, the depletions of prey animals (base foods), illegal trades, and conflicts with local people have caused further declines in their wild populations. The snow leopard is therefore assigned “Endangered C1” in The 2010 International Union for Conservation Nature and Natural Resources Red List of Threatened Animals [4]. The snow leopard is also now listed in Appendix I in the Convention on International Trade in Endangered Species of Wild Fauna and Flora [5]. Before 1974, the number of animals introduced from the wild in world captive population was greater than animals born in captivity. However, since 1974, this situation has been reversed [6]. In Japan, of all the 26 captive animals on December 31, 2009, only one animal was born in the wild [7]. It is therefore imperative to breed captive populations of snow leopards, with the animal's reproductive physiology needing to be resolved for successful breeding managements.

Several scientific reports have shown that female snow leopards are seasonally polyestrous in winter [8]. Their estrous periods are from January to May, with delivery of from 1 to 5 cubs occurring from April to June in the wild [1]. In Japan under captivity, the parturitions are usually seen between March and August (mostly in May) [7]. However, the basic reproductive physiology of this endangered wild cat is currently almost unknown.

Fecal sampling requires no specialized equipment or facilities and is considerably easier than drawing blood or sampling urine [9]–[11]. Although serum steroid hormones are the most accurate reflection of ovarian hormone secretion, repeated blood samplings are impractical in non-tractable animals such as non-domestic felids. It has also been shown in felids that steroid metabolites are excreted almost exclusively in feces, with very little found in the urine. For example, injection of radio-labeled steroids into domestic or non-domestic cats showed that >85% of metabolites were excreted in the feces within 1–2 days [10], [12], [13]. Therefore, fecal hormone analysis is the preferred method for long-term monitoring of reproductive physiology in non-domestic felids [9]–[11].

These studies imply that noninvasive fecal steroid metabolite analysis is an effective method of monitoring endocrine function in felids. The serum estradiol-17β (E_2_) and progesterone (P_4_) concentrations in female snow leopards were reported by Schmidt *et al*. [8] and Roth *et al*. [14]. Brown *et al*. [10] also suggested that changes in serum E_2_ and P_4_ concentrations were reflected in fecal E_2_ and P_4_ metabolite concentrations in this species.

Fecal cortisol metabolite concentration has been reported in various felids, except snow leopard [13], [15]–[20]. The most common method used in these reports is a commercially available corticosterone radioimmunoassay (RIA). Unfortunately, this method cannot be used in zoos or aquariums, as RIA requires the use of radioisotopes. Recently, cortisol enzyme immunoassay (EIA) has proved effective for measuring fecal corticoids in carnivore species [17], [20]. Longitudinal profiles of both cortisol EIA and corticosterone RIA were found to be qualitatively similar, with the data being highly correlated. This suggests that both systems were equally effective for monitoring adrenal activity [15]. In this study, fecal E_2_, P_4_, and cortisol metabolite concentrations were monitored using an EIA method in captive pregnant and pseudopregnant female snow leopards.

Recently, we reported that individual housing was better than group housing, and longer separation was highly advantageous for breeding success in female cheetahs (*Acinonyx jubatus*) [21]. The snow leopard is a solitary cat like the cheetah and therefore we expect that individual housing may also result in increased libido in female snow leopards. In order to estimate the effect of housing conditions on reproductive endocrinology, we changed the conditions of one female from that used for the other two females. Although there is no scientific evidence, it is generally accepted that the best breeding successes is achieved by individual housing.

## Results

### 2.1. Daily fecal sex steroid hormone metabolite concentrations

**Figure 1:**
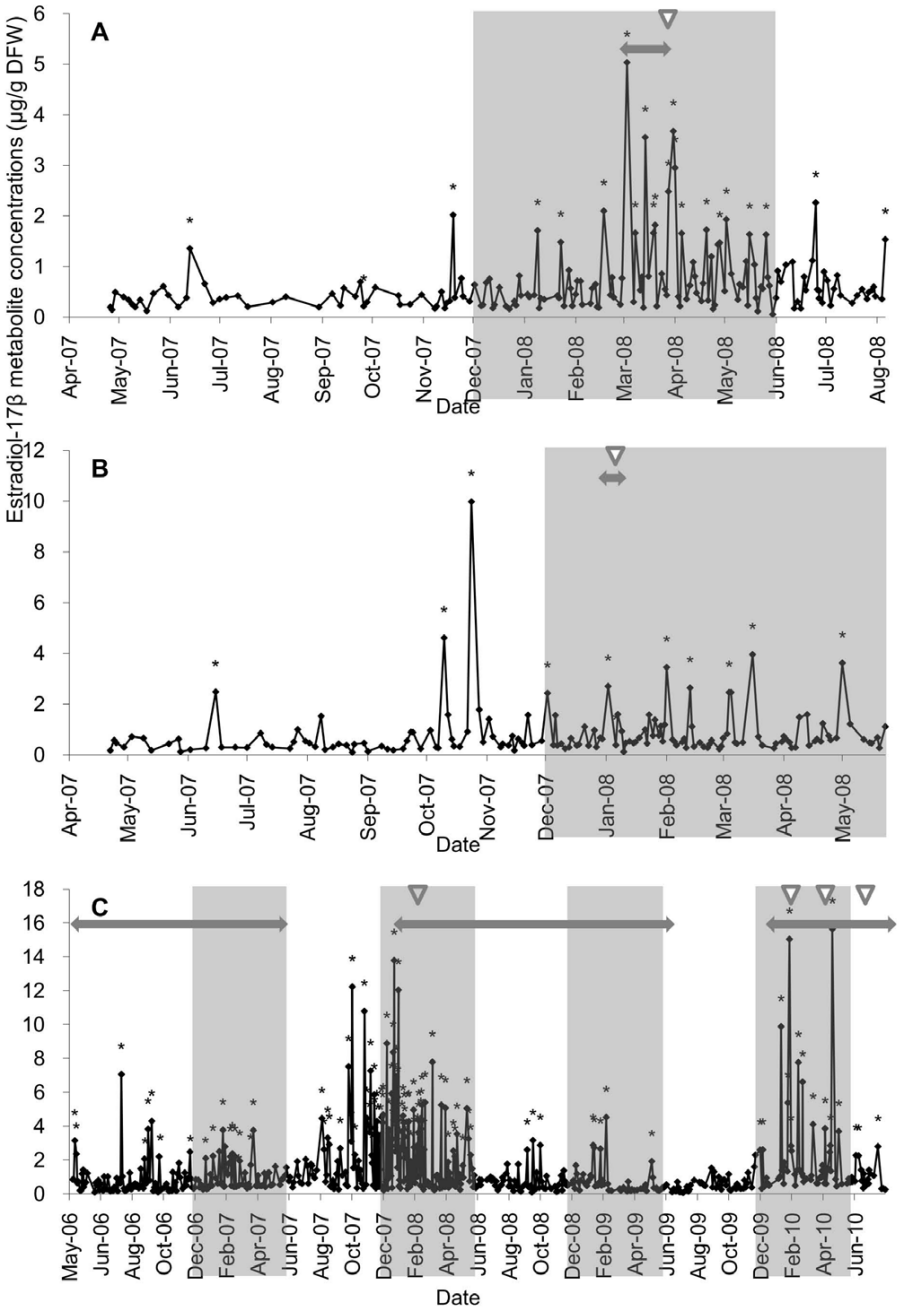
Daily fecal estradiol-17β metabolite concentrations in three female snow leopards (A: female A, B: female B, C: female C). The shaded areas represent the breeding season, (i.e., December to May). The two-headed arrows show the period that the female was housed with a male, white arrowheads show the day of last copulation. The asterisks indicate the estradiol-17β metabolite peaks.

Figure 1 depicts the fecal E_2_ metabolite profiles in the three female animals. The E_2_ metabolite concentrations ranges were 0.05–5.03 in female A (n = 174), 0.09–9.98 in female B (n = 155), and 0.04–13.05 µg/g DFW in female C (n = 588). During the breeding season, E_2_ metabolite concentrations in female C were significantly higher than in females A and B (*P*<0.05). Female A copulated with a male (#2178) from March 17 to 26, 2008 including the day of E_2_ metabolite peak. Female A became pregnant and normally delivered a cub on June 26, 2008. Female B copulated with the same male from January 2 to 6, 2008 including the day of E_2_ metabolite peak, and normally delivered three cubs on April 9, 2008. Female C copulated with a male (#2452) from March 2 to 9, 2008 and from February 12 to 15, April 1 to 9 and June 23 to 24, 2010 including the day of E_2_ metabolite peak. This copulation by female C resulted in pseudopregnancy, which culminated in two returns of estrous in 2010.

**Figure 2:**
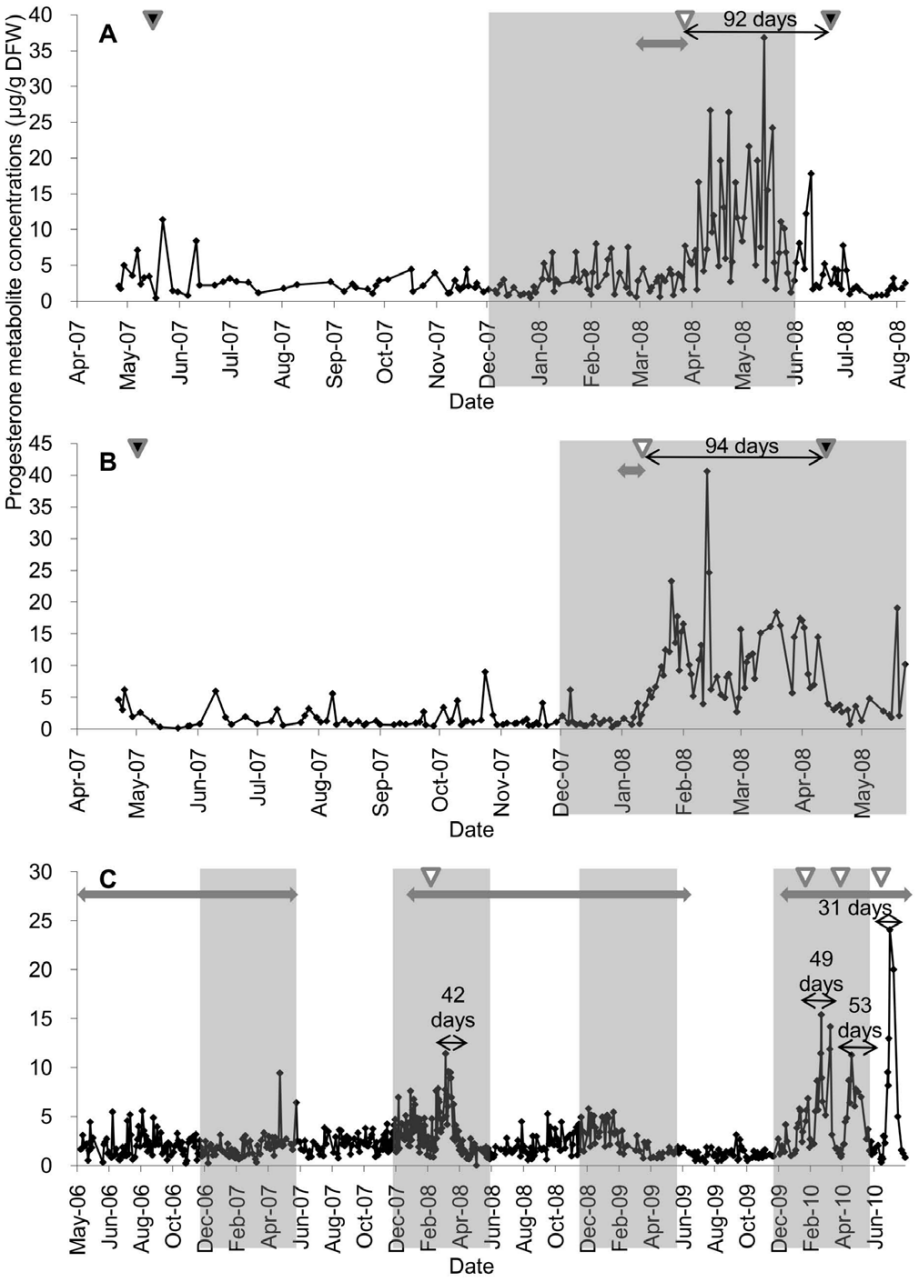
Daily fecal progesterone metabolite concentrations in three female snow leopards (A: female A, B: female B, C: female C). The shaded areas, two-headed arrows and white arrowheads are explained in Fig.1. The black arrowheads designate the day of parturition. The thin two-headed arrows and the number of days represent the pregnancy or pseudopregnancy duration.

The P_4_ metabolite profiles including the pregnant and pseudopregnant periods are shown in Figure 2. These concentration ranges were 0.40–36.85 in female A (n = 174), 0.15–40.61 in female B (n = 154) and 0.21–24.75 µg/g DFW in female C (n = 593). In females A and B, the periods of pregnancy were 92 days and 94 days, respectively. During this period, P_4_ metabolite concentration increased significantly to 36.85 in female A and 40.61 µg/g DFW in female B. On the other hand, female C had four pseudopregnant periods with approximately durations of 42, 49, 53 and 31 days. These periods were approximately one-third or one-half of the pregnant periods in females A and B. During these pseudopregnant periods, P_4_ metabolite concentration increased significantly to 11.58 (the pseudopregnancy in 2008), 15.84 (1^st^ pseudopregnancy in 2010), 11.75 (2^nd^ pseudopregnancy in 2010) and 24.75 µg/g DFW (3^rd^ pseudopregnancy in 2010). These values were considerably lower than those measured in females A and B during pregnancy.

### 2.2. Daily fecal cortisol metabolite concentrations

**Figure 3:**
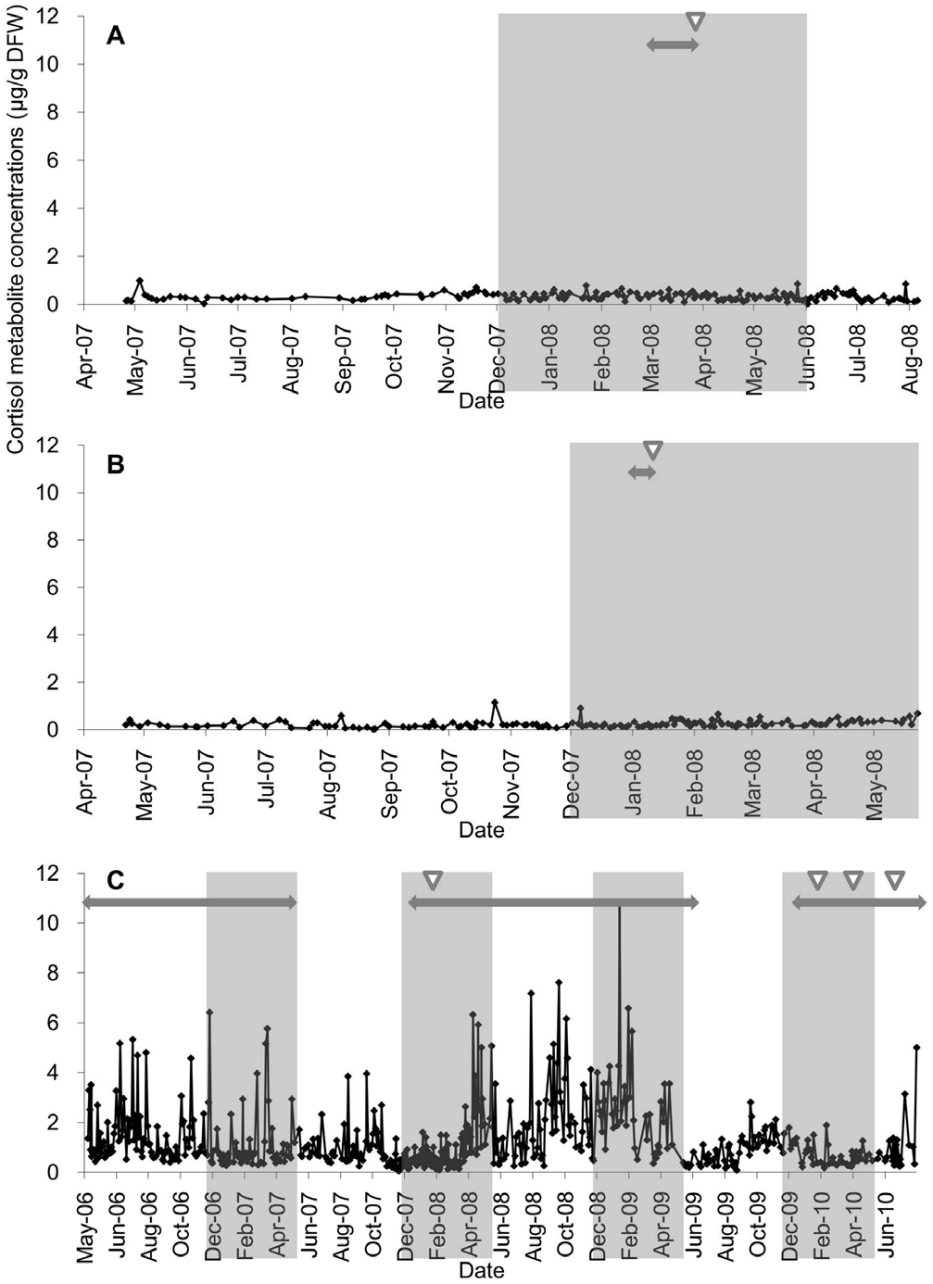
Daily fecal cortisol metabolite concentrations in three female snow leopards (A: female A, B: female B, C: female C). The shaded areas, two-headed arrows, and white arrowheads are explained in Fig.1.

Figure 3 shows the fecal cortisol metabolite profiles in the three female animals. The cortisol metabolite concentrations ranges were 0.01–0.99 in female A (n = 175), 0.02–1.15 in female B (n = 155), and 0.05–10.77 µg/g DFW in female C (n = 596). Throughout this study, the cortisol metabolite concentrations in female C who failed to get pregnant on four occasions were significantly higher than in females A and B who succeeded in becoming pregnant (*P*<0.05).

### 2.3. Effect of housing conditions on steroid hormone metabolite levels

**Figure 4:**
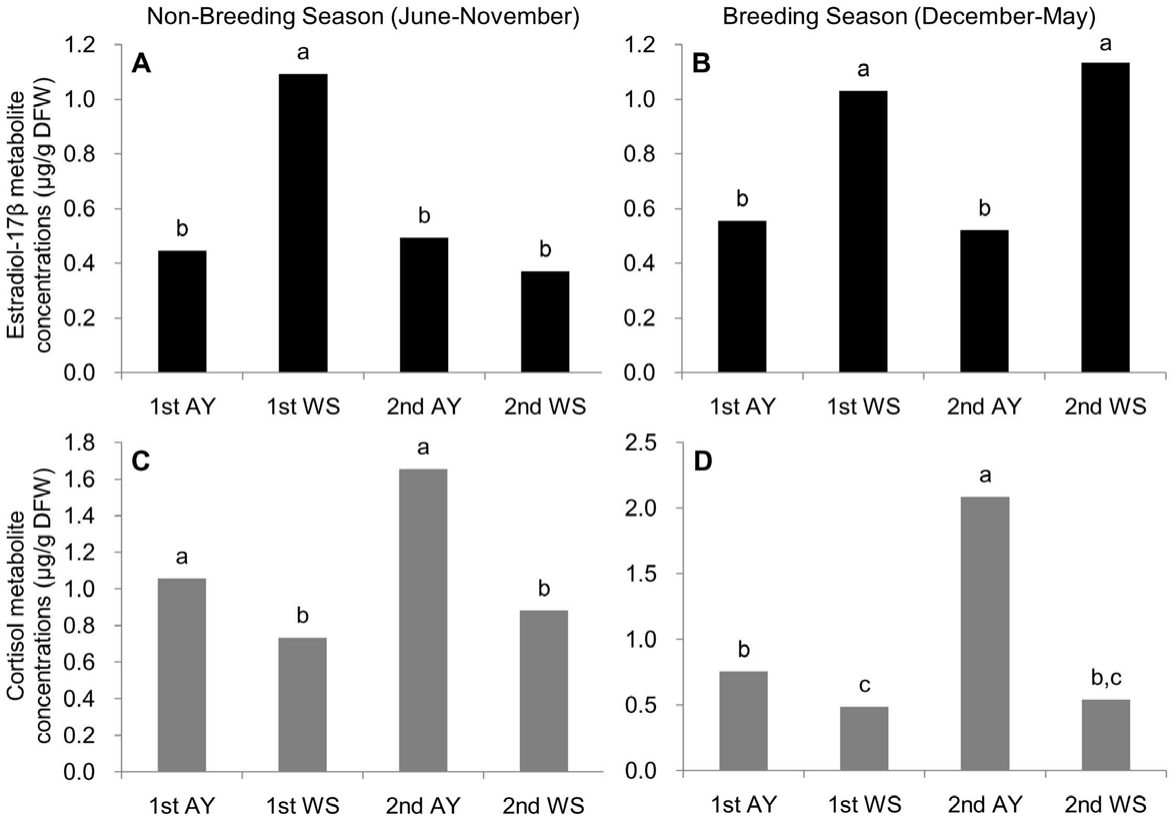
Median values of fecal estradiol-17β (A: non-breeding season, B: breeding season) and cortisol metabolite concentrations (C: non-breeding season, D: breeding season) in female C. AY and WS are abbreviations for the style of housing condition, AY: living all-year with a male (1^st^ and 2^nd^ AY are the years 2006 and 2008), WS: living only during the winter season with a male (1^st^ and 2^nd^ WS are the years 2007 and 2009).

Data collected over a 4-year period of the C, E_2_, and cortisol metabolite concentrations in the female animals were compared between breeding and non-breeding seasons and between the two different types of housings (AY, with a male all year and WS, with a male only in winter) (see methods for detailed housing). Fig.4A shows that during non-breeding seasons, there was no significant difference (*P*<0.05) in E_2_ metabolite concentration between AY and WS (1^st^ AY: 0.45, 0.08–7.05 µg/DFW; 2^nd^ AY: 0.50, 0.07–3.25 µg/DFW; 1^st^ WS: 1.09, 0.24–12.22 µg/DFW; 2^nd^ WS: 0.37, 0.05–15.87 µg/DFW). However, during breeding seasons, E_2_ concentrations in the 1^st^ AY (median value, 0.56; range, 0.25–3.76 µg/DFW) and 2^nd^ AY (0.52, 0.13–4.51 µg/DFW) were significantly lower (*P*<0.05) than those in the 1^st^ WS (1.03, 0.20–13.78 µg/DFW) and 2^nd^ WS (1.13, 0.30–15.63 µg/DFW) (Fig.4B).

In contrast to the changes in E_2_ metabolite concentrations during the non-breeding seasons, cortisol metabolite concentrations in the 1^st^ AY (1.06, 0.40–5.33 µg/DFW) and 2^nd^ AY (1.66, 0.25–7.62 µg/DFW) were significantly higher (*P*<0.05) than those in the 1^st^ WS (0.73, 0.21–3.96 µg/DFW) and 2^nd^ WS (0.54, 0.18–1.89 µg/DFW) (Fig.4C). However, during breeding seasons there was no significant difference (*P*<0.05) in cortisol metabolite concentrations between AY and WS (1^st^ AY: 0.76, 0.27–6.41 µg/DFW; 2^nd^ AY: 2.09, 0.33–10.77 µg/DFW; 1^st^ WS: 0.49, 0.05–6.33 µg/DFW; 2^nd^ WS: 0.54, 0.18–1.89 µg/DFW) (Fig.4D).

## Discussion

This study examined endocrinological changes under different housing conditions in female snow leopards. Our results showed that living together during the winter season (WS style) was the most suitable housing method for promoting reproduction. From our findings, individual housing during non-breeding seasons should increase successful captive breeding. To our knowledge this is also first report is on long-term monitoring of fecal P_4_ and cortisol metabolite concentrations in pregnant and pseudopregnant female snow leopards.

In this study, P_4_ metabolite concentrations remained elevated for 30 to 50 days during pseudopregnancy and for about 90 days during pregnancy. It is well known that the gestation period of pregnancy is about 90–100 days, although we found no report of changes in P_4_ concentration during pregnancy. There are two reports on P_4_ concentrations in pseudopregnant snow leopards. Brown *et al*. [10] showed that fecal P_4_ concentrations were maintained at a high level for at least 60 days, while Schmidt *et al*. [8] reported that high serum P_4_ concentrations were measured for 6 weeks. These periods were of similar duration to those in the present study. We should also note that the duration of pseudopregnancy in this species was approximately one-third or one-half of the duration of pregnancy, similar to that seen in other felids [10], [15].

It is well known that female and male snow leopards usually live alone in the wild, except during breeding seasons. Female cheetahs are also solitary wild cats. Meltzer [22] suggested that female cheetahs must be housed with male cheetahs only during periods in which they exhibit estrous behavior in order to avoid habituation to the males. If these animals were housed permanently together, the libido of both females and males decreased markedly. Meltzer also observed that breeding success was often triggered by intermittent contact between females and males. Wielebnowski [23] and Wielebnowski and Brown [24] reported that unpaired cheetahs were more likely to breed successfully. Moreover, we reported that a female cheetah that had been separated from the other cheetahs for a long time was the only female that succeeded in breeding. However, in the study, we found that the hormone profiles were similar in all the females, with estrogen peaks occurring at regular intervals of approximately 8 to 15 days, regardless of the different housing conditions [21]. Thus, long term separation appears to be almost necessary for successful breeding in cheetahs, although hormonal changes may not be involved.

In the current study, female C mated with a male only if the opportunities for meeting were only provided in the winter season. These housing conditions are similar to those in the wild. E_2_ metabolite concentrations, with this WS style housing during the breeding season being significantly higher than those with AY style housing. From these results, we suspect that the libido of female snow leopards increased when they were housed during the breeding season only. This is clearly shown by the change in E_2_ metabolite concentrations observed in this study. Furthermore, although not statistically significant, the cortisol metabolite concentrations with the AY style housing during the breeding season were comparatively higher than those with WS style housing. As cortisol reflects stress levels, these results may imply that stress is a factor that decreases libido in females during the breeding season. Note that the decrease in libidos was indicated by the E_2_ concentrations with AY style housing, and showed no marked increase even during the breeding season.

Another finding in this study was that cortisol metabolite the concentrations with AY style housing during the non-breeding season were also significantly higher than those with WS style housing. In clouded leopards (*Neofelis nebulosa*), lower levels of fecal corticoids were seen in individuals housed in enclosures with more vertical space, while higher corticoid levels were found in individuals housed in close proximity to other large predators [25]. It has also been shown that oncilla (*Leopardus tigrinus*) females exhibited distinct elevations in fecal corticoid concentrations after being transferred from large enriched enclosures to smaller barren cages, with the concentrations reducing when the animals were moved back to an enrichment cage [26].

These results of corticoids should be related to territory formation. Fundamentally, animals form their own territory that suits their lifestyle. Snow leopards are solitary during non-breeding seasons, but live together with the opposite sex only during breeding seasons. Because of this seasonal change in social behavior the animals form territories that are different in size and place depending on the season [27]. In the current study, female C could have formed a territory narrower than normal when she had been housed with a male in the non-breeding season. This may have resulted in higher stress in the animal that was indicated by high levels of cortisol metabolites. Our results implied that housing with a male during the non-breeding season is stressful to female snow leopards.

In this study, the cortisol metabolite concentrations in female C were significantly higher than those in females A and B. Chronic stress induces longitudinal secretion of glucocorticoids and is known to inhibit normal reproductive function [20], [24], [25]. In the spotted hyena (*Crocuta crocuta*), it was reported that plasma cortisol concentrations in nulliparous females were usually higher than those in parous females [28]. Female C who failed to became pregnant in the current study had higher cortisol metabolite levels than females A and B who gave births successfully. This occurred in spite of significantly higher E_2_ metabolite concentrations in female C during the breeding seasons than in females A and B. Although the reproductive capacity of the males was not examined, these high cortisol levels may be a major factor that inhibited pregnancy in female C.

Recently, the risk of extinction has become highly relevant for the snow leopard. It is, therefore, extremely important to save animals in zoos without restocking from the wild. This study examined the endocrinology changes in female snow leopards under different housing conditions and showed the importance of housing condition to reproductive success. The current findings should contribute to an increase in the reproductive efficiency of these animals under captivity.

## Materials and Methods

This experiment was performed as part of reproductive trials of species (Project Wild Cats of the Japanese Study Group on Artificial Reproduction of Endangered Animals) in the Kobe Municipal Oji Zoo and the Tama Zoological Park. The objective of these trials was to save these species and promote animal welfare: hence, no permission was necessary. This study complied with applicable national laws.

### 4.1. Animals

#### 4.1.1. Female A

Female A (International Studbook #2168) was kept at the Tama Zoological Park, and had been monitored for 1.5 years from April 26, 2007 to August 6, 2008. This female had been housed with a male (#2178) only during the winter season from March 12 to 28, 2008. She had a normal delivery of a cub on May 19, 2007 at the start of this study.

#### 4.1.2. Female B

Female B (#2068) was also kept at Tama Zoological Park and was monitored for almost one year from April 22, 2007 to May 23, 2008. As for female A, this female was housed with a male (#2178) only during the winter season from January 2 to 8, 2008. Female B had a stillborn cub on May 2, 2007 at the start of this study.

#### 4.1.3. Female C

Female C (#2482) was nulliparous and was kept at Kobe Municipal Oji Zoo. This female was monitored for about four years from May 8, 2006 to August 7, 2010. She was housed with a male (#2452) in two different housing styles. The first style was living together all the year (termed “AY”) (1^st^ and 2^nd^ AY conducted in the years 2006 and 2008). The second style involved the animals living together only during the winter season (termed “WS”) (1^st^ and 2^nd^ WS conducted in the years 2007 and 2009). As for the WS style, female C was housed with the male during the winter season when estrous behaviors was observed frequently (flehmen, sniffing, rubbing, rolling, prusten, spraying, *etc.*) [29]. In details, this involved female C being housed with the male from the start of this study to June 21, 2007 (1^st^ AY), from January 16, 2008 to June 30, 2009 (from the breeding season of 1^st^ WS to the end of 2^nd^ AY), and from December 30, 2009 to the finish of this study (the breeding season of 2^nd^ WS).

All the snow leopards, including the male mate, were in visual, olfactory, and auditory contact with other animals through adjacent cages. At night (basically around 5:00 PM–9:00 AM), the individual animals were housed in their own room. Depending on the zoo, all the snow leopards were fed mainly horseflesh and bones of poultry or rabbits supplemented with vitamins and minerals. The animals were kept fasting for 1 day a week. Water was available *ad libitum*.

### 4.2. Fecal steroid hormone analysis

Fecal samples were collected at least three or more times a week, and stored at −80°C until analysis. The feces were dried in an electric oven (DRA330DA, Advantec Toyo Kaisya Ltd., Tokyo, Japan) at 50°C for 24 h and then pulverized. A 0.06 g sample of powdered feces was vortexed for 30 min in 3 ml of 80% methanol, and then centrifuged at 2,500 rpm for 10 min. Steroid standards were added to each sample. The mean recovery rates of each steroid hormone from fecal samples were: E_2_, 99.5%; P_4_, 99.0%; cortisol, 95.2% (n = 5).

The extracts of E_2_, P_4_ and cortisol metabolite in the supernatant were analyzed by enzyme-immunoassay (EIA) according to methods described elsewhere [29]. Briefly, the fecal extracts was diluted 8-fold with EIA buffer (0.15-M NaCl, 0.04-M Na_2_HPO_4_, 0.1% bovine serum albumin, pH 7.2) and duplicate 20 µl aliquots of this solution added to 96-well plates bound with goat anti-rabbit IgG (H+L) (Code#270335, Seikagaku Biobusiness Co., Tokyo, Japan). To obtain the standard curve, 0.195–100 ng/ml E_2_ (052–04041; Wako Pure Chemical Industries, Ltd., Osaka, Japan), P_4_ (P-8783; Sigma-Aldrich Co., Steinheim, Germany), or cortisol (H4001; Sigma-Aldrich Co.) diluted with EIA buffer were also dispensed into the wells in duplicate. Immediately after the addition of 100 µl E_2_ (6.5∶8000, FKA236E; Cosmo Bio Co., Ltd., Tokyo, Japan), P_4_ (5.7∶8000, FKA302E; Cosmo Bio Co., Ltd., Tokyo, Japan), or cortisol antiserum (10∶8000, FKA404E; Cosmo Bio Co., Ltd., Tokyo, Japan) and an equal volume of horseradish peroxidase conjugated E_2_ (13∶8000, FKA235; Cosmo Bio Co., Ltd., Tokyo, Japan), P_4_ (8∶8000, FKA301; Cosmo Bio Co., Ltd.) or cortisol (8∶8000, FKA403; Cosmo Bio Co., Ltd., Tokyo, Japan), the plates were incubated in the dark for 12 h at 4°C. Free-bound separation was achieved by emptying the plate and washing four times with 0.05% Tween-80 solution. A mixture of 75 µl substrate buffer solution A (0.01-M urea hydrogen peroxido, 0.1-M Na_2_HPO_4_, 0.05-M citric acid) and 75 µl of solution B (0.002-M 3,3′,5,5′,-tetra methyl benzidine, 4% dimethyl sulfoxide, 0.05-M citric acid) was added to each well, followed by incubation for 8–15 min at 37°C in the dark. The reaction was stopped by the addition of 4-N H_2_SO_4_ (50 µ1) and the absorbance at 450 nm measured using a microplate reader (Model 550, BIO-RAD Laboratories Inc., Tokyo, Japan). The values for all the steroids were expressed as the means of duplicate determinations, corrected for extraction recovery. They are indicated as µg/g of dry fecal weight (DFW).

The polyclonal E_2_ antiserum (FKA236E; Cosmo Bio Co., Ltd., Tokyo, Japan) was raised in rabbits against 6-oxo-estradiol-3-CME-BSA and cross-reacted with E_2_ 100%, estradiol-3-glucuronide 56.3%, estradiol-3-sulfate 26.8%, estrone-3-glucuronide 1.2%, estrone-3-sulfate 0.86%, estrone 0.8%, estriol 0.5%, and testosterone 0.05%. The polyclonal P_4_ antiserum (FKA302E; Cosmo Bio Co., Ltd.) was raised in rabbits against progesterone-3-CMO-BSA and cross-reacted with P_4_ 100%, 5α-pregnanedione 12.5%, 11α-OH-progesterone 5.3%, pregnenolone 2.0%, 20α-OH-progesterone 0.2%, and 0.01% with deoxycorticosterone, 17α-OH-progesterone, corticosterone, cortisol, and aldosterone. The polyclonal cortisol antiserum (FKA404E; Cosmo Bio Co., Ltd.) was raised in rabbits against cortisol-3-CMO-BSA and cross-reacted with cortisol 100%, 11-deoxvcortisol 11.5%, cortisone 4.0%, 17α-hydroxy-11-deoxy-corticosterone 0.2%, 17α-hydroxy-progesterone 0.04%, and 0% with the other steroids.

A recovery test was conducted by adding each steroid standard to a subset of sample extracts. The mean recovery of each steroid hormone from the fecal extracts were: E_2_, 107.2%; P_4_, 98.8%; and cortisol, 99.7% (n = 8). Parallel displacement curves were obtained by comparing serial dilutions of pooled fecal extracts and the each steroid standard preparation. The sensitivity of the assays were 0.2, 1.2, and 3.9 pg/well for E_2_, P_4_, and cortisol respectively. The intra- (5-well within a plate) and inter-assay (12 plates) coefficients of variation were 8.5% and 16.5% for E_2_, 5.6% and 3.9% for P_4_, and 1.6% and 5.3% for cortisol.

### 4.3. Statistical analysis

Data are presented as mean value ± SEM or median value (range minimum-maxim value).

For each female, the baseline E_2_ and P_4_ metabolite concentrations were calculated using an iterative process in which values that exceeded 2 standard deviations (SD) above the mean were excluded. The average was then recalculated and the elimination process repeated until no values exceeded 2SD above the mean [10], [30]. Based on the method reported by Pelican *et al*. [31], the average of the remaining values was considered as the “baseline” for the animals. Values greater than 3 times the baseline were considered “elevated.” The pregnant or pseudopregnant period was defined from the last mating day to the delivery day or the day that P_4_ metabolite concentration declined to the baseline level.

The Steel-Dwass multiple comparison method was used to test for differences in E_2_ and cortisol concentrations between the female animals. This method was carried out using a statistical analysis program which was operated by the Genome Information Research Center of Osaka University on the Web (http://www.gen-info.osaka-u.ac.jp/testdocs/tomocom/). Furthermore, E_2_ or cortisol metabolite concentrations in each non-breeding and breeding season were also compared using the Steel-Dwass multiple comparison method in order to evaluate the effect of different housing conditions on the dynamics of steroid hormone concentration. The offsprings were born in Japan from March to August [7]. This data, coupled with the known gestation of 90–100 days, were used to identify December to May as the period of most successful matings. These months were therefore designated as the “breeding season,” and the period from June to November as the “non-breeding season” in this study. A *P* value of <0.05 was deemed significant.
